# Clinical Profile of Hepatoblastoma: Experience From a Tertiary Care Centre in a Resource-Limited Setting

**DOI:** 10.7759/cureus.26494

**Published:** 2022-07-01

**Authors:** Arkadeep Dhali, Tuhin S Mandal, Somak Das, Gautam Ray, Prasenjit Halder, Debarshi Bose, Suparna K Pal, Sukanta Ray, Abhijit Chowdhury, Gopal Krishna Dhali

**Affiliations:** 1 Department of Gastrointestinal Surgery, School of Digestive and Liver Diseases, Institute of Postgraduate Medical Education and Research, Kolkata, IND; 2 Department of Pediatric Gastroenterology, School of Digestive and Liver Diseases, Institute of Postgraduate Medical Education and Research, Kolkata, IND; 3 Department of Hepatology, School of Digestive and Liver Diseases, Institute of Postgraduate Medical Education and Research, Kolkata, IND; 4 Department of Radiation Oncology, Institute of Post Graduate Medical Education and Research, Kolkata, IND; 5 Department of Gastroenterology, School of Digestive and Liver Diseases, Institute of Postgraduate Medical Education and Research, Kolkata, IND

**Keywords:** chemotherapy, resource-limited setting, outcome, surgery, hepatoblastoma

## Abstract

Background

Hepatoblastoma (HB) is a rare neoplasm of the liver, accounting for about 1% of all pediatric cancers. The aim of the present study is to report our experience with HBs over a period of five years from a tertiary center in Eastern India.

Methodology

This is a retrospective observational study. The data of all patients who were diagnosed with HB between August 2015 and December 2020 was reviewed.

Results

Twenty-three patients who were diagnosed and treated for HB at our center were included in the study. Sixteen (69.5%) of them were male. The median age of presentation was 14 (range, 3-58) months. An abdominal lump (n=23, 100%) and abdominal pain (n=11, 47.8%) were the most common presenting symptoms. The median level of serum alpha-fetoprotein at the time of initial evaluation was 8000 (878-1,280,000) ng/dL. The mean size of the largest focus in its largest dimension was 12.03±3.77 cm. The epithelial variant (n=22, 95.7%) was the most common histological subtype. One (4.3%), 10 (43.4%), 11 (47.8%), and one (4.3%) patient were found to have pre-treatment extent of tumor (PRETEXT) stages 1, 2, 3, and 4, respectively. Fifteen (65.2%) children were classified as standard risk and seven (34.7%) children as high risk.

All the patients received neoadjuvant chemotherapy (NACT). The most commonly performed surgery was right hepatectomy (n=12, 52.1%). There were three (13%) cases of perioperative mortality. Four postoperative complications developed in three (13%) patients. Four (17.3%) patients developed chemotherapy-related complications. The median duration of follow-up was 31 (range, 0-58) months. Three (13%) patients developed relapses of the disease. Overall, five-year survival in our series was 73.9%.

Conclusion

This study shows that the overall outcomes of HB in a resource-limited setting such as ours are good with the adoption of multi-modality treatment. Managing chemotherapy-induced complications and making liver transplantation more feasible will improve the results further.

## Introduction

Hepatoblastoma (HB) is a rare neoplasm of the liver with an estimated incidence of 1.5 cases per a million children, accounting for about 1% of all pediatric cancers [1]. The global annual incidence of all childhood cancers exceeds 200,000, of which more than 80% are from the developing world [2]. In India, it has not been a priority. In addition to this, limited financial resources, difficulty in accessing healthcare, and lack of awareness contribute to the late stages of presentation [3].

HB is generally seen below five years of age with a male predominance [4]. Maternal smoking, alcohol, or oral contraceptive use, prematurity, and low birth weight are some of the associated risk factors. It is also often associated with syndromes like Li-Fraumeni syndrome, Beckwith-Weidmann syndrome, familial adenomatous polyposis, and trisomy 18 [4]. Surgical intervention after neoadjuvant chemotherapy (NCT) is the accepted line of treatment with only a subset of patients who can undergo upfront surgery. The feasibility of resection of the liver should be individually assessed as it may be associated with a high risk of recurrence. The aim of the present study is to report our experience with HBs over a period of five years from a tertiary center in Eastern India.

## Materials and methods

This is a retrospective observational study. Data of all patients who were diagnosed with HB between August 2015 and December 2020 were retrieved from our prospectively maintained pediatric gastroenterology database. Demographic details, clinical and laboratory parameters, treatment details, follow-up data, and outcomes were reviewed. This study was approved by the institutional ethics committee (Memo number: IPGME&R/RAC/312, dated 16/4/2022). The need for informed patient consent was waived by the ethics committee as the data was anonymized and the retrospective nature of the study. This study is registered at the Thai Clinical Trials Registry and was conducted in accordance with the Declaration of Helsinki (TCTR20220418005). The study has been reported in line with the STROCSS criteria [5].

Diagnosis

The diagnosis of HB was made on the basis of clinical features, characteristic imaging features, elevated serum alpha-fetoprotein (AFP) levels, and histology. The pre-treatment extent of tumour (PRETEXT) stage of the disease was assigned based on contrast-enhanced computed tomography (CECT) of the abdomen, pelvis, and chest [6]. The liver is divided into four sectors: left lateral, left medial, right anterior, and right posterior (corresponding to Couinaud segments II/III, IV, V/VIII, and VI/VII, respectively) [6,7]. The PRETEXT system is utilized by all major study groups [6]. Based on the PRETEXT stage [stages I-IV], additional anatomical involvement (M: distant metastasis; V: vena cava or all three hepatic veins involvement; E: extrahepatic contiguous spread; P: portal vein bifurcation, main portal vein or both portal veins involvement; R: tumour rupture; N: positive lymph nodes), and the AFP level, the patients are classified as "Standard Risk (SR)" [PRETEXT stages I-III with AFP > 100 ng/ml] or "High Risk (HR)" [PRETEXT IV; any PRETEXT with AFP < 100 ng/ml; any PRETEXT with additional anatomical involvement as detailed above] [8]. Core tissue biopsy was done for all patients and the pathological diagnosis of HB was established along with histological sub-categorization. Management options were decided by a multidisciplinary team comprising of pediatric gastroenterologists, gastrointestinal (GI) surgeons, gastroenterologists, hepatologists, oncologists, GI radiologists, and GI pathologists.

Treatment

In our institution, we follow the International Society of Pediatric Oncology - Liver Tumor Strategy Group (SIOPEL) guidelines, which have also been endorsed by the Indian Council of Medical Research (ICMR) for India [8]. The chemotherapy regime for SR patients is four cycles of NACT comprising Cisplatin monotherapy administered every three weeks (80 mg/m^2^ infusion over 24 hours, along with pre and post-hydration) and two cycles of the same after surgery [8]. The choice of surgical intervention was based on the anatomical distribution of the lesion. The initial chemotherapy regimen of choice was PLADO (Cisplatin and Doxorubicin), but following the study by Perilongo et al. showing similar complete resection and survival rates with cisplatin monotherapy vis-a-vis PLADO, monotherapy is now followed as chemotherapy complications are minimized [7,9]. For HR patients, the SIOPEL-3 guidelines are followed, wherein cisplatin (80 mg/m^2^ as above) is given for one cycle, followed by carboplatin-doxorubicin (carboplatin 500 mg/m^2^ IV over 1 hour and doxorubicin 60 mg/m^2^ IV over 48 hours) for the next cycle [10]. Such patients are given a total of ten cycles (seven neoadjuvant cycles and three post-resection), with each cycle given two weeks apart. Granulocyte colony-stimulating factor (G-CSF) is used as per guidelines [10]. All patients in the HR group underwent surgical resection after the first seven cycles of chemotherapy. Criteria for liver transplantation have been advocated by the study groups and include multifocal PRETEXT IV disease, portal vein or all three hepatic veins involvement, and insufficient downgrading post NACT, among others [11]. At our institution, given the excellent response of HB to chemotherapy, along with the fact that most families have economic constraints in carrying on with prolonged immunosuppression following transplantation, we administer NACT for all stages and then reassess. Chemotherapy regimens for recurrent or non-responsive diseases have not been well crystallized in the literature, with irinotecan having been used, among others [12]. In our institution, we individualize therapy in such situations based on consultation with the oncologist.

Statistical analysis

Quantitative variables were expressed as mean ± standard deviation or median with range. Dichotomous variables were expressed as a percentage. Survival analysis was done using the Kaplan Meier method. All statistical computations were done using IBM SPSS, version 20 (IBM Corp. Chicago, USA).

## Results

During our present study period, 23 patients were diagnosed with hepatoblastoma and were included in the study. Sixteen (69.5%) of them were male. Two (8.6%) of the patients were born prematurely, and none had any syndromic features. The median age of presentation was 14 (range, 3-58) months. The median age of diagnosis of the disease was 16 (7-60) months. The duration of symptoms ranged from 0.2 to 3 (median = 1) months. Abdominal pain (n=11, 47.8%) was the most common presenting symptom, followed by fever (n=8, 34.7%) and weight loss (n=8, 34.7%). Five (21.7%) patients developed a loss of appetite and one (4.3%) developed a cough. None of the patients had jaundice. On physical examination, pallor and an abdominal lump were found in all (n=23, 100%) patients.

The mean hemoglobin level was 7.83±1.47 g/dL and the mean platelet count was 4.45±2.77 lakh/mL. The median level of serum alpha-fetoprotein at the time of initial evaluation was 8000 (878-1,280,000) ng/dL. One (4.3%) patient had seropositivity for the hepatitis C virus. A contrast-enhanced computed tomography of the chest, abdomen, and pelvis was done to stage the disease (PRETEXT). One (4.3%), 10 (43.4%), 11 (47.8%), and one (4.3%) patient were found to have PRETEXT stages 1, 2, 3, and 4, respectively. The right lobe of the liver was involved in most of the patients (n=11, 47.8%), followed by the involvement of both lobes (n=8, 34.7%) and the left lobe (n=4, 17.3%). Most of the patients (n=12, 52.1%) had involvement in two sectors. Others had involvement of three (n=9, 39.1%), four (n=1, 4.3%), and one sector (n=1, 4.3%). The median number of segments of the liver involved was four (range, 1-7). Most of the patients (n=20, 86.9%) had a unifocal disease. Three (13.1%) of the patients had a multifocal disease. The mean size of the largest focus in its largest dimension was 12.03±3.77 cm. Involvement of all hepatic veins (V) was seen in one (4.3%) patient. Portal vein involvement (P) was demonstrated in four (17.3%) patients. Two (8.6%) patients developed spontaneous rupture (R) of tumors. Two (8.6%) patients had bilateral pulmonary metastasis.

The epithelial variant (n=22, 95.7%) was the most common histological subtype observed in our series, followed by mixed epithelial and mesenchymal variants (n=1, 4.3%). Among the 22 patients with epithelial variants, pure fetal variety was seen in 18 (81.8%) patients, followed by mixed fetal and embryonal variety in two (9%) patients, pure embryonal variety in one (4.5%) patient, and small cell undifferentiated subtype in one (4.5%) patient.

According to the SIOPEL risk stratification system, 15 (65.2%) children were classified as standard risk, and seven (34.7%) children as high risk. All the patients had normal vestibular, cardiac, and renal function at the start of treatment. All the patients received NACT. The median number of cycles of NACT given was five (range, 4-8). The most common regime of chemotherapy given was cisplatin monotherapy (n=10, 43.4%), followed by SUPER PLADO (n=8, 34.7%), and PLADO (n=5, 21.7%). Four (17.3%), 17 (73.9%), and two (8.6%) patients were found to have POSTTEXT stages 1, 2, 3, and 4, respectively. The (mean) size of the largest liver lesion on the preoperative CT scan was 6.59±2.88 cm.

The median age at the time of surgery was 20 (10-66) months. Makuuchi type (n=9, 39.1%) was the most common type of incision applied during surgery, followed by right subcostal type (n=7, 30.4%), transverse type (n=17.3%), and Chevron incision (n=3, 13.04%). The types of surgical interventions performed are presented in Table 1. The most commonly performed surgery was right hepatectomy (n=12, 52.1%). The median operating time was 230 (210-301) minutes. The median intraoperative blood loss was 250 (100-500) ml. Two (8.6%) patients required intraoperative blood transfusions. Microscopic free margins could be achieved in 13 (56.5%) patients. There were three (13%) cases of perioperative mortality. One (4.3%) patient died due to intraoperative cardiac arrest secondary to arrhythmia. One (4.3%) patient developed uncontrolled bleeding intraoperatively from the right hepatic artery adhered to the tumor during non-anatomical wedge resection of the liver. One (4.3%) patient died intraoperatively due to extensive bleeding from rupture of the tumor. Four postoperative complications developed in three (13%) patients. The most common complication was surgical site infection (n=3, 13%), followed by bile leak (n=1, 4.3%). All the complications were managed conservatively.

**Table 1:** Details of surgical procedures (n=23)

Type of surgery	n	%
Primary surgical procedure for removal of tumor		
Right hepatectomy	12	52.1
Non-anatomical wedge resection	5	21.7
Extended right hepatectomy	2	8.6
Left hepatectomy	1	4.3
Extended left hepatectomy	1	4.3
Right lateral segmentectomy	1	4.3
Left lateral segmentectomy	1	4.3
Additional surgical procedure		
Right adrenalectomy	1	4.3

**Figure 1:**
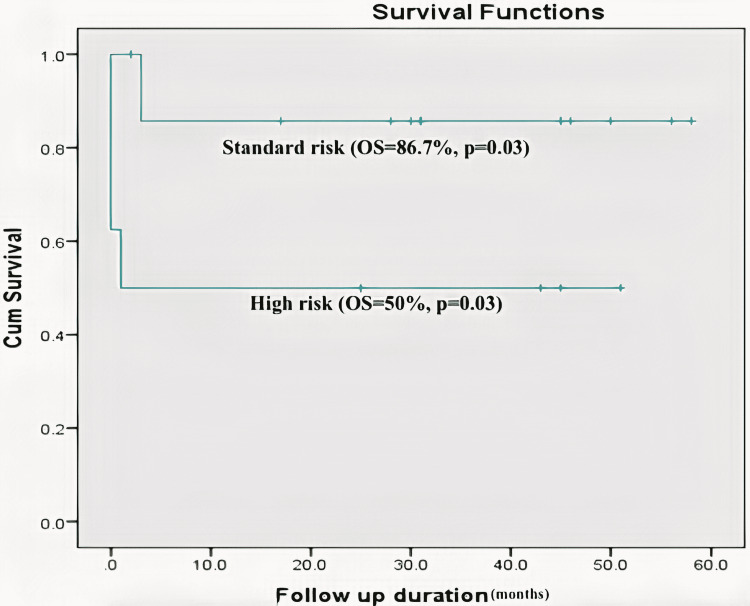
Kaplan Meier curve showing survival in standard risk and high risk patients (p=0.03)

The median number of cycles of postoperative chemotherapy given was two (range, 0-3). Four (17.4%) patients developed chemotherapy-related complications (pre-op=2, post-op=2). Tissue necrosis (n=4, 17.4%) was the most common chemotherapy-induced complication, followed by febrile neutropenia in 3 (13%) and anorexia complicated by severe acute malnutrition in one (4.3%) patient. One of the patients who developed febrile neutropenia developed sepsis and succumbed to the illness. The median duration of follow-up was 31 (range, 0-58) months. Three (13%) patients developed relapses of the disease. Two of those who developed relapse at three-month follow-up succumbed to the disease despite receiving adjuvant chemotherapy. Overall survival in our series was 73.9%. The patients with standard risk had a higher rate of overall survival (13/15, 86.7%) compared to those with higher risk (4/8, 50%) (Figure 1).

## Discussion

Very few recent studies on hepatoblastoma (HB) from the Indian subcontinent have been published [13,14]. Older published data lack key information like PRETEXT staging and histopathological variants. This has limited our understanding from the overall clinicopathological point of view. In a resource-limited setting, diagnosis of HB is often a challenge owing to a lack of representative data. Most of the recent studies are based on Western literature, where the availability of resources is different.

HB is a rare pediatric hepatic tumor, mainly affecting children between six months and three years of age. Male children are more frequently affected. In our study, the mean age of presentation was 14 months, and 69.5% of the study participants were male. This was similar to other contemporary studies [14,15]. In another Indian study by Manuprasad et al., female predominance was reported [3].

The presentation of HB is nonspecific. Most of the patients present with vague upper abdominal discomfort or pain [3]. Other symptoms are anorexia, weight loss, nausea, and vomiting. Similarly, in our study, nonspecific upper abdominal pain (47.8%) was the most common presentation, and pallor (100%) and palpable lump (100%) were the most common physical findings.

Major advances in the clinical management of HB have been made due to studies by the International Childhood Liver Tumor Strategy Group (SIOPEL), which recommends the PRETEXT system of assessment and the use of neoadjuvant chemotherapy followed by surgery and adjuvant chemotherapy. This has made significant improvements in the treatment and outcomes for patients diagnosed with HB, and the survival rate is reaching 80% [16,17]. The majority of our patients had PRETEXT stage II (n=10, 43.4%) or III (n=11, 47.8%). This is similar to the SIOPEL-1 study [7]. The SIOPEL group has shown in a randomized trial that children having standard risk when given six-cycles of cisplatinum monotherapy had similar outcomes to those who were treated with combination therapy of cisplatinum (cumulative dose of 480 mg/m2) and doxorubicin [9]. Moreover, this strategy has reduced the occurrence of chemotherapy-induced toxicities [7,9]. Ototoxicity [18], secondary to cisplatin, may be inversely related to age at the time of exposure, where very young patients exhibited a higher degree of hearing loss [19]. In a randomised, controlled trial by SIOPEL-6, compelling evidence suggested that sodium thiosulfate administered six hours post-cisplatin administration resulted in a lower degree of hearing loss among children with standard-risk without jeopardizing event-free survival (EFS) or overall survival (OS) [20].

In Children’s Oncology Group (COG) study AHEP 0731, children having very low-risk (stage I, pure fetal histological variant) didn’t receive any chemotherapy, low-risk patients received only two cycles of cisplatin + fluorouracil + vincristine (C5V) postoperatively, and intermediate-risk patients received neoadjuvant chemotherapy with two to four courses of C5V along with doxorubicin (30 mg/m2) [21]. The COG recommends upfront surgery followed by adjuvant chemotherapy. The outcomes of both COG and SIOPEL are comparable. In our institution, we follow the SIOPEL guidelines, which have also been endorsed by the Indian Council of Medical Research (ICMR) for India [8]. In our center, all (100%) patients underwent surgical resection after receiving neoadjuvant chemotherapy. In other series, authors have reported cases of unresectable tumors due to the progression of diseases in spite of neoadjuvant therapy [3,14,22]. At our institution, given the excellent response of HB to chemotherapy, along with the fact that most families have economic constraints in carrying on with prolonged immunosuppression following transplantation, we administer neoadjuvant chemotherapy for all stages and then reassess. Hence, none of the patients underwent liver transplantation at our center, even though the facility exists. In a recent study at a dedicated liver transplant center, Shanmugam et al. showed that 63% of the patients could undergo surgical resection and 20% required liver transplantation [14]. Around 13% of our patients had relapses of the disease. The overall survival (OS) was 73.9%, which was comparable to other contemporary studies [3,13,14]. Chemotherapy-induced toxicity was minimal in our series compared to other studies [3,13,14]. This may be due to the use of single-agent cisplatin instead of PLADO.

None of the patients in our series failed to follow up. Hence, we could detect long-term treatment-related toxicity.

The study has some strengths and limitations. The strength is that it is one of the largest single-center case series on HB reported from India. Considering the rarity of the tumor, the sample size was decent with acceptable long-term outcomes. The drawbacks are: (1) it is a retrospective study spanning over a period of five years; (2) it is purely a surgical series where only resection was performed and no comparison was made between other modalities of surgery (transplant); and (3) it is a single-center experience. Our experience shows that a combination of chemotherapy and surgery offers good long-term results.

## Conclusions

The present study outlines that even in resource-limited settings, with the adoption of a multi-disciplinary strategy, clinical outcomes of hepatoblastoma are good. In experienced hands, surgery can be performed with acceptable perioperative morbidity and mortality and excellent long-term outcomes. In our study, none of the patients underwent liver transplantation due to the unavailability of resources, even though adequate expertise exists. Making liver transplantation more accessible will further improve the overall outcome.

## References

[1] López-Terrada D, Alaggio R, de Dávila MT (2014). Towards an international pediatric liver tumor consensus classification: proceedings of the Los Angeles COG liver tumors symposium. Mod Pathol.

[2] Barr R, Riberio R, Agarwal B, Masera G, Hesseling P, Magrath I (2006). Pediatric oncology in countries with limited resources. Principles and Practice of Pediatric Oncology.

[3] Manuprasad A, Radhakrishnan V, Ramakrishnan AS (2018). Hepatoblastoma: 16-years’ experience from a tertiary cancer centre in India. Pediatr Hematol Oncol J.

[4] Reynolds P, Urayama KY, Von Behren J, Feusner J (2004). Birth characteristics and hepatoblastoma risk in young children. Cancer.

[5] Mathew G, Agha R (2021). STROCSS 2021: strengthening the reporting of cohort, cross-sectional and case-control studies in surgery. Int J Surg.

[6] Couinaud C (1954). [Liver lobes and segments: notes on the anatomical architecture and surgery of the liver]. Presse Med (1893).

[7] Pritchard J, Brown J, Shafford E (2000). Cisplatin, doxorubicin, and delayed surgery for childhood hepatoblastoma: a successful approach--results of the first prospective study of the International Society of Pediatric Oncology. J Clin Oncol.

[8] Agarwala S, Gupta A, Bansal D (2017). Management of hepatoblastoma: ICMR consensus document. Indian J Pediatr.

[9] Perilongo G, Maibach R, Shafford E (2009). Cisplatin versus cisplatin plus doxorubicin for standard-risk hepatoblastoma. N Engl J Med.

[10] Zsíros J, Maibach R, Shafford E (2010). Successful treatment of childhood high-risk hepatoblastoma with dose-intensive multiagent chemotherapy and surgery: final results of the SIOPEL-3HR study. J Clin Oncol.

[11] Gupta AA, Gerstle JT, Ng V (2011). Critical review of controversial issues in the management of advanced pediatric liver tumors. Pediatr Blood Cancer.

[12] Zsíros J, Brugières L, Brock P (2012). Efficacy of irinotecan single drug treatment in children with refractory or recurrent hepatoblastoma--a phase II trial of the childhood liver tumour strategy group (SIOPEL). Eur J Cancer.

[13] Archana B, Thanka J, Sneha LM, Xavier Scott JJ, Arunan M, Agarwal P (2019). Clinicopathological profile of hepatoblastoma: an experience from a tertiary care center in India. Indian J Pathol Microbiol.

[14] Shanmugam N, Scott JX, Kumar V (2017). Multidisciplinary management of hepatoblastoma in children: experience from a developing country. Pediatr Blood Cancer.

[15] Arora RS (2012). Outcomes of hepatoblastoma in the Indian context. Indian Pediatr.

[16] Craddock N, Dlova N, Diedrichs PC (2018). Colourism: a global adolescent health concern. Curr Opin Pediatr.

[17] Meyers RL, Maibach R, Hiyama E (2017). Risk-stratified staging in paediatric hepatoblastoma: a unified analysis from the Children's Hepatic tumors International Collaboration. Lancet Oncol.

[18] Dall'Igna P, Brugieres L, Christin AS (2018). Hepatoblastoma in children aged less than six months at diagnosis: a report from the SIOPEL group. Pediatr Blood Cancer.

[19] Yancey A, Harris MS, Egbelakin A, Gilbert J, Pisoni DB, Renbarger J (2012). Risk factors for cisplatin-associated ototoxicity in pediatric oncology patients. Pediatr Blood Cancer.

[20] Brock PR, Maibach R, Childs M (2018). Sodium thiosulfate for protection from cisplatin-induced hearing loss. N Engl J Med.

[21] Katzenstein HM, Furman WL, Malogolowkin MH (2017). Upfront window vincristine/irinotecan treatment of high-risk hepatoblastoma: a report from the Children's Oncology Group AHEP0731 study committee. Cancer.

[22] Singh T, Satheesh CT, Appaji L, Aruna Kumari BS, Padma M, Kumar MV, Mukherjee G (2010). Hepatoblastoma: experience from a single center. Indian J Cancer.

